# Teaching mass casualty incident management to senior medical students by three-dimensional tabletop exercise without lecture

**DOI:** 10.1186/s12909-025-07434-x

**Published:** 2025-06-05

**Authors:** Wei-Kuo Chou, Ming-Tai Cheng, Chien-Hao Lin

**Affiliations:** Department of Emergency Medicine, National Taiwan University Hospital, No.7, Chung Shan S. Rd. (Zhongshan S. Rd.), Zhongzheng Dist., Taipei City, 100225 Taiwan, Republic of China

**Keywords:** Mass casualty incident, Tabletop exercise, Three-dimensional, Emergency department, Disaster medicine

## Abstract

**Background:**

Traditional methods of teaching mass casualty incident (MCI) management often lack engagement, particularly for senior medical students. Lectures may be uninspiring, and tabletop exercises (TTX) may not fully captivate participants. This study proposes the use of three-dimensional (3D) models in TTX as a solution to these challenges.

**Methods:**

A TTX focusing on MCI in the emergency room was developed for senior medical students, centered on five core competencies of MCI management. 3D models representing the emergency room, hospital staff, patients, and other personnel were utilized. No lectures were given; instead, students engaged in discussions and demonstrated responses using 3D models. Immediate feedback was provided by the instructor, and knowledge was tested through multiple-choice questions and questionnaires.

**Results:**

Between September 2018 and May 2022, 326 students completed pre- and post-exercise evaluations. Significant improvements were observed in test scores for all core competencies, as well as increased interest in learning and willingness to participate. Students found the exercise engaging and expressed a desire for further training.

**Conclusions:**

A TTX using 3D models is proving to be an effective alternative of teaching MCI management and disaster medicine to senior medical students, while increasing interest and participation.

**Supplementary Information:**

The online version contains supplementary material available at 10.1186/s12909-025-07434-x.

## Background

Understanding disaster medicine is vital for successful disaster response, particularly for mass casualty incidents (MCIs). To ensure that health care systems operate efficiently in the setting of a disaster or MCI, medical students need to develop core competencies of emergency preparedness, incident command, and their roles in disaster response [1, 2]. Following proper education, these future physicians may serve as a resource to support disaster management [3]. Moreover, most medical students are willing to respond in case of disasters [3]. Therefore, it is important to provide all future physicians with the fundamental knowledge to understand and respond to threats posed by disasters [1]. Nevertheless, previous reports indicate that they do not have adequate education regarding disaster medicine or management [1, 3]. It is therefore important to develop an education method of disaster medicine targeting medical students.

Since disasters are not common events, using disaster response experience for educational purposes is not practical [4]. Disaster medicine is traditionally taught by lectures and tested by exercises. While earlier reviews questioned the consistency of evidence regarding educational strategies for MCIs, more recent studies have demonstrated that various methods—including high-fidelity simulation, tabletop exercises (TTX), and blended learning—can effectively enhance learners’ knowledge, confidence, and preparedness for MCI scenarios [5]. Since learning is a process in which knowledge is created by the transformation of experiences, disaster exercises may be a method for teaching disaster medicine [6]. Exercises are considered as an opportunity to introduce concepts of disaster medicine and challenges experienced during hospital MCI to participants [7]. However, the most commonly used teaching methods are full-scale exercises (FSEs) and lectures rather than TTXs [7]. Although FSEs are valuable for MCI education, they are often complex, costly, and resource-intensive. For educational programs operating with limited resources, implementing FSEs may be impractical without substantial preparation and support. In contrast, TTX offer a feasible and scalable alternative that can provide meaningful experiential learning at a lower cost [7, 8].

TTX is another potential approach [9]. Findings from other studies indicate that TTX is effective in enhancing the competency-related knowledge and skills of participants [6, 10]. Another study also revealed that TTX training methods are more effective than conventional lectures [11]. The flexibility and adaptability of TTXs can be enhanced by adding multiple events or injects and information that advance the story or scenarios which participants are dealing with. These are also essential features in the design of educational tools [11].

Previous studies have shown that TTXs can improve participants’ confidence and perceived preparedness for disaster response. However, their effectiveness in enhancing objective knowledge remains uncertain [8, 11]. In many studies demonstrating the effectiveness of TTXs, participants received prior lectures or assigned readings. Didactic lectures may reduce student engagement and motivation, especially among medical students already burdened with heavy memorization workloads. [2, 12] In contrast, TTXs offer experiential learning in a more informal and interactive format, which can enhance participation and promote deeper exploration of disaster medicine [11]. In these cases, the evaluation of exercise effectiveness may have been influenced by the teaching that preceded the exercise [13, 14].

Alternatively, typical TTX are limited to verbal discussions supported by slide presentations, occasionally supplemented with static images or video clips. This approach may weaken the effectiveness of TTXs by insufficiently clarifying the situation. To overcome this problem, TTX using board format has been employed. This approach was associated with good learning outcomes, but remains rarely used with students and is more complex to design [9].

We hypothesized that the use of three-dimensional (3D) modelling in TTX may enhance teaching effectivity, and that this approach can be used directly for teaching without the need for pre-exercise training or classes. The aim of this study was to introduce, develop, and evaluate a new disaster medicine teaching tool for medical students.

## Methods

### Study setting and design

We conducted a prospective observational study involving final-year medical students who participated in an emergency medicine internship at National Taiwan University Hospital (NTUH) between July 2018 and May 2022. NTUH is a 2,700-bed academic medical center that provides both primary and tertiary care. During the study period, a total of 495 final-year medical students completed the internship across 60 rotations. The study was approved by the Institutional Review Board of the NTUH (REC No. 201905098RIND), and informed consent was provided by all students prior to enrolment.

### The three-dimensional tabletop exercise

#### The design

One of the key innovations in our disaster medicine training program was the use of a three-dimensional tabletop exercise (3D-TTX) as the primary instructional tool. Notably, students participated in the 3D-TTX without receiving any prior didactic lectures on MCI management.

The 3D-TTX was specifically developed to simulate a hospital-based response to an MCI and was incorporated into the emergency medicine internship curriculum. To introduce students to the fundamentals of emergency medicine and hospital-based MCI management, a scenario was designed based on a real-life incident—an explosion at a train station near the hospital [15]. The scenario simulated the arrival of 23 patients to the emergency department (ED) of National Taiwan University Hospital (NTUH) within a one-hour period, including five casualties having major trauma. The exercise was intentionally designed to replicate resource constraints and create a sense of being overwhelmed, thereby challenging students to prioritize tasks and manage patient surge under pressure.

Prior to designing the 3D-TTX, a needs analysis was conducted by three instructors from the disaster medicine training program. All instructors were emergency physicians with over 15 years of experience in disaster medicine education at NTUH. The needs analysis involved a three-round consensus process and a literature review on disaster medicine core competencies (DMCCs) [1, 16–18]. An initial list of 10 DMCCs was identified. After evaluating their applicability to a TTX format, their relevance to immediate emergency response, participants’ medical backgrounds, and time limitations, five core competencies were selected for emphasis in the 3D-TTX: incident management system, recognition, notification, and initiation, patient triage, surge capacity and capability and recovery and demobilization.

A total of 18 injects were developed and introduced sequentially along a timeline to reflect these five competencies. The design of the 3D-TTX was informed by established international guidelines, including the World Health Organization (WHO) Simulation Exercise Manual and the Homeland Security Exercise and Evaluation Program (HSEEP) [19]. A master scenario events list (MSEL) was finalized to guide the structured implementation of the exercise. (Appendix A) (Table 1).

**Table 1:** Summary of exercise injects

Inject No	Description	Corresponding Core Competency
1	Fire department informs ED of train station bombing; possible mass casualties expected	Recognition, Notification, and Initiation
2	Emergency personnel report to the hospital; roles need to be assigned	Incident Management System
3	Plan and designate emergency treatment zones	Incident Management System
4	Three walking wounded patients self-present to the ED	Patient Triage
5	Ambulance brings 15 casualties to the ED	Surge Capacity and Capability
6	Hospital leadership inquires about activating additional emergency measures	Surge Capacity and Capability
7	Additional emergency personnel arrive; expansion of response team required	Incident Management System
8	Family brings in obviously deceased victim, requests resuscitation	Patient Triage
9	Patient initially able to walk suddenly collapses and deteriorates	Patient Triage
10	Multiple family members arrive at ED searching for patients	Surge Capacity and Capability
11	Ambulance brings pulseless patient with no visible injuries	Surge Capacity and Capability
12	Family members call ED requesting patient information	Surge Capacity and Capability
13	Journalists rush into the ED attempting to interview patients	Incident Management System
14	Public health authority calls for hospital status update	Incident Management System
15	Journalists demand a press conference from hospital	Incident Management System
16	Fire department informs ED that all victims have been transported	Recovery and Demobilization
17	All severely injured patients are hospitalized; only 5 mild cases remain	Recovery and Demobilization
18	Hospital administration asks about post-response activities	Recovery and Demobilization

**Fig. 1:**
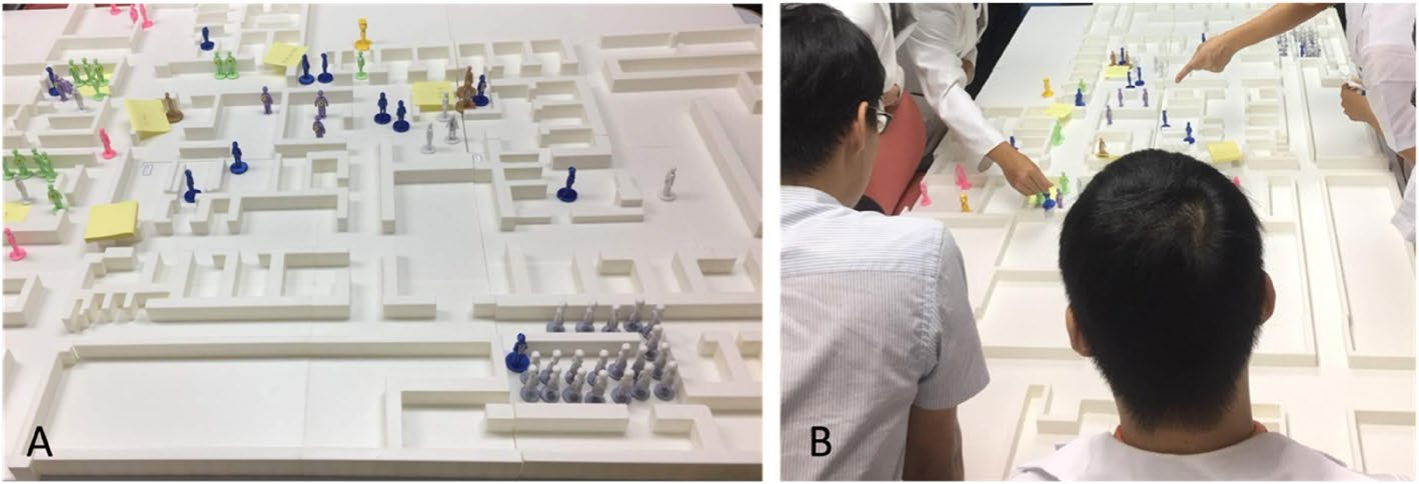
Setting up and conducting the tabletop exercise (TTX). **A** Miniature model of a hospital emergency department and roles in that setting. **B** Conducting the TTX based on a holistic ‘bird’s eye’ view

Another innovative feature of the 3D-TTX was the creation of a miniature physical model of the hospital ED, extended treatment areas, and other relevant operational zones using 3D printing (Fig. 1A). To enhance participants’ understanding of the spatial layout and improve situational awareness, the 3D model was fully integrated into the exercise design. Miniature figurines were employed to represent various key roles, including hospital staff, patients, family members, and journalists. Additional patient information and scenario-specific injects were presented through slides, complementing the physical setup. This combined approach was intended to support visualization of dynamic interactions between patients and responders, facilitate comprehension of complex scenarios, and promote an immersive, experiential learning environment (Fig. 1B).

### The implementation

**Fig. 2:**
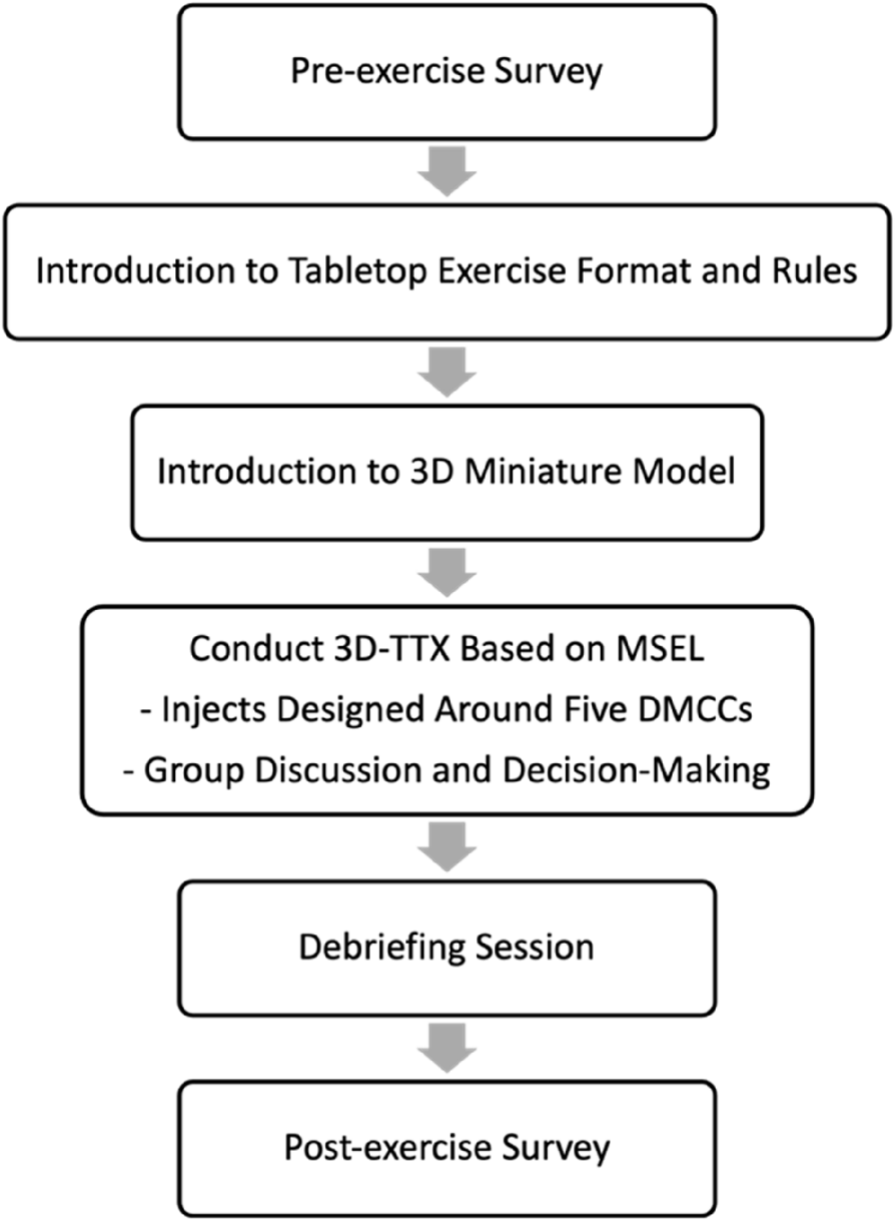
Flowchart of the implementation process of the 3D-TTX

The implementation process of the 3D-TTX is illustrated in Fig. 2. At the beginning of the exercise, the miniature ED model was arranged to reflect the normal operational status of the ED. One of the three instructors from the disaster medicine training program facilitated the session.

The exercise scenario, along with a series of injects derived from the pre-identified core competencies, was delivered sequentially by the facilitator according to the guidance provided by the MSEL. Miniature figurines corresponding to each event were placed on the 3D model to simulate real-time developments. For example, patient figurines were added to the ED to represent patient arrivals during the MCI. For each inject, students were prompted to discuss appropriate response strategies and implement their decisions directly on the 3D model. The facilitator engaged participants in reflective questioning, asking them to explain the rationale behind their actions and to articulate the reasoning that informed their decisions. Students were also encouraged to ask questions regarding the advantages and disadvantages of various response strategies and to clarify hospital-specific guidelines and protocols. Following each discussion, the facilitator highlighted the relevant DMCCs associated with the inject and introduced the fundamental concepts in disaster medicine. Students were then invited to reflect, revise, or reaffirm their decisions based on this newly acquired knowledge, thereby reinforcing the DMCCs through active learning.

The exercise concluded once all injects had been addressed. The total duration of the activity was approximately two hours. A debriefing session followed the exercise, during which the facilitator summarized key learning points and responded to additional questions raised by the participants.

### Recruitments

The exercise was conducted for all students who selected the emergency medicine internship as part of their undergraduate medical training. Each rotation involved a group of approximately 7 to 11 students. Participants worked together as a single group, collaboratively discussing and responding to each scenario inject throughout the session. While all participating students completed the exercise and associated evaluations, they were given the option to consent to the use of their evaluation data for research and analysis. Only data from students who provided informed consent were included in the final analysis.

### Outcomes

The outcome measures of this study included: interest in learning disaster medicine, willingness to participate in MCI management, and knowledge of DMCCs.

Prior to the exercise, participants completed a pre-exercise survey to collect demographic information and background knowledge. The data included age, gender, previous disaster training experience (PDTE), interest in learning disaster medicine, willingness to participate in MCI management, and performance on a knowledge assessment (Appendix B). Interest in disaster medicine and willingness to participate in MCI management were measured using self-assessment items on a 5-point Likert scale (1 = very uninterested/unwilling; 5 = very interested/willing). Knowledge was assessed using a 10-item multiple-choice questionnaire, with each correct answer awarded 10 points and incorrect answers receiving zero points. Two questions were developed to assess each of the five selected DMCCs. The knowledge assessment was designed and validated by the three primary instructors and two additional disaster medicine instructors. Prior to the study, the internal consistency of the assessment was evaluated in a pilot test among 32 ED nurses, yielding a Cronbach’s alpha of 0.83.

Following the 3D-TTX, participants completed a post-exercise survey using the same instrument to evaluate changes in interest, willingness, and knowledge. Additionally, a self-assessment of perceived learning effectiveness was conducted to evaluate the subjective improvement, using a 5-point Likert scale (1 = very ineffective; 5 = very effective). The post-exercise survey also included an open-ended section for participants to provide qualitative feedback on the training experience.

The design of the knowledge assessment was directly informed by the structured development process of the 3D-TTX, which included objective setting, competency selection, and MSEL development. The evaluation approach followed the exercise planning cycle recommended by the HSEEP and was reviewed by subject-matter experts in disaster medicine. Accordingly, the assessment tools were tailored to align with the specific competencies and operational context emphasized in the training.

### Statistical analysis

A paired *t*-test was used to analyse the differences in the mean scores before and after the exercise. The measured data were described as mean ± standard deviation (SD). A *p*-value < 0.05 was considered to indicate a statistically significant difference. Data were analysed using SPSS version 20.0 (IBM Corp., Armonk, NY, USA).

## Results

During the study period, a total of 495 senior medical students participated the emergency medicine internship. Of these, 326 students consented to participate the study and completed both pre- and post-exercise surveys, resulting in a response rate of 65.9%.

The demographic characteristics of the participants are presented in Table 2. The mean age was 24.01 years (standard deviation [SD] = 1.30 years). Among the respondents, 74.2% were male, and 73.0% reported no previous disaster training experience (PDTE).

**Table 2:** Demographic characteristics of participants

Characteristics	Participants (*n* = 326)
**Age in years, mean (SD)**	24.0 (1.3)
**Gender**	
Male, *n* (%)	Male: 242 (74.2)
Female, *n* (%)	Female: 84 (25.8)
**PDTE, number of event (%)**	
0	238 (73.0)
1	71 (21.8)
2	16 (4.9)
3	1 (0.3)

*PDTE* Previous disaster training experience

The results of the outcome measures are summarized in Table 3. There was a significant increase in participants’ self-reported interest in learning disaster medicine following the 3D-TTX (mean score: 4.25 post-exercise vs. 4.17 pre-exercise; *p* < 0.001). Similarly, participants’ willingness to participate in MCI management also improved significantly after the exercise (4.43 vs. 4.29; *p* = 0.013).

**Table 3:** Scores of the pre- and post-exercise for interest in learning disaster medicine, willingness to participate in MCI management, and knowledge assessment

Outcome measures	Pre-exercise Mean (SD)	Post-exercise Mean (SD)	*p*-value
**Interest in learning disaster medicine**	4.17 (0.74)	4.25 (0.67)	< 0.001
**Willingness to participate in MCI management**	4.29 (0.76)	4.43 (0.69)	0.013
**Knowledge assessment**	44.85 (19.88)	75.69 (11.50)	< 0.001
Incident management system	3.46 (3.81)	9.60 (5.54)	< 0.001
Recognition, notification, and initiation	8.92 (4.81)	13.88 (4.39)	< 0.001
Patient triage	9.89 (5.87)	16.74 (3.55)	< 0.001
Surge capacity/capability	13.06 (6.36)	18.26 (2.82)	< 0.001
Recovery and demobilization	9.49 (6.08)	17.19 (3.78)	< 0.001

*MCI* Mass casualty incident, *SD* Standard deviation

In terms of knowledge acquisition, the total scores on the knowledge assessment were significantly higher in the post-exercise survey compared to the pre-exercise survey (mean score: 75.69 vs. 44.85; *p* < 0.001). In addition, scores related to each of the five DMCCs showed a consistent and statistically significant increase (Table 3).

The participants’ self-assessment of learning effectiveness following the exercise is presented in Table 4. The vast majority of students (96.9%) reported a subjective improvement (score = 4 or 5) in their understanding of disaster medicine after participating in the 3D-TTX.

**Table 4:** Student self-assessment of learning effectiveness through exercise

Rating	*N* (%)
1 – Very ineffective	0 (0)
2 – Ineffective	0 (0)
3 – Neutral	4 (1.2)
4 – Effective	73 (22.4)
5 – Very effective	243 (74.5)
Missing	6 (3.8)
Total	326 (100)

In the open-ended response section of the post-exercise survey, many participants regarded the 3D-TTX as a highly effective tool for enhancing their understanding and knowledge of disaster medicine. One participant stated, "I gained a clearer understanding of mass casualty management." Another commented, "It was interesting and also helped everyone grasp the structure of the entire system, making us less likely to panic." Students frequently noted that the use of 3D miniature models added a sense of realism to the exercise, making the learning experience more engaging and immersive. Several comments emphasized that the visual and spatial representation of the emergency department facilitated a clearer understanding of the overall response process and decision-making dynamics during a MCI.

### Data availability statement

The datasets used and analysed during the current study are available from the corresponding author on reasonable request.

## Discussion

Various educational strategies have been used to teach medical students disaster medicine, including traditional ones (e.g., lectures and case studies) and less established ones (e.g., TTXs and drills) [2]. To our knowledge, this is the first published description of a curriculum that utilizes 3D miniature models to teach disaster medicine and hospital MCI management to medical students without requiring any pre-exercise reading or lectures. This represents an innovative adaptation of the TTX format and demonstrates its feasibility and suitability as an engaging instructional method for undergraduate medical education.

Traditional didactic lectures, while useful for delivering foundational knowledge, often fall short in engaging learners in disaster medicine, particularly due to the rarity of real disaster experiences of the participants. Disasters are complex, high-stakes events that are difficult to conceptualize without firsthand exposure. This limits learners’ ability to grasp the cognitive and procedural demands of disaster response. TTX is an alternative way of presenting scenarios to participants. However, traditional TTX lacks realism and the means to display the information [11].

Moreover, disasters and MCIs are complex events involving multiple patients, dynamic operational demands, and various environmental factors, which are difficult to simulate within the constraints of a traditional classroom setting [20]. Medical students, unlike practicing physicians, have minimal clinical exposure and are even less likely to have encountered real-world disaster scenarios [21]. To overcome this problem, scenarios should be used in disaster medicine education of medical students.

Three-dimensional miniature models may be a solution to this problem. In this context, our study highlights the potential of the 3D-TTXs to bridge this gap by creating a more immersive and imaginative learning environment.

The use of 3D miniature models in TTX offers several advantages. Firstly, participants can see and experience the scenario more directly. The 3D miniatures can be used to represent elements involved in an MCI response and can be moved according to the participant’s decision [22]. Standing by the table, participants can see the whole picture of response, allowing for a longer span of attention for all participants. [6] Compared to conventional TTX that rely on textual or two-dimensional visual aids, the incorporation of 3D-printed models allows learners to contextualize scenarios within a familiar hospital setting. In our study, most students reported that the 3D-TTX was both engaging and helpful for understanding disaster medicine and MCI response. These findings suggest that the use of 3D miniature models may serve as a visually stimulating element that captures students’ attention—an observation supported by verbal feedback and student engagement noted during the exercise. It is important to acknowledge that visual appeal was not formally measured in the current evaluation. The 3D miniature models provide opportunities for participants to experience a disaster environment and respond to incidents as they would in a real-life case [11]. This physical representation enhances spatial reasoning and situational awareness, facilitating a deeper understanding of logistical constraints, resource allocation, and team coordination under crisis conditions.

Secondly, through this method, participants can realistically experience the function of the system over time and the evolution of their role in it [2]. The bird’s eye view enabled all participants to observe every aspect of the sequence and roles played in the exercise, thus benefiting maximally [2]. This advantage provides visual feedback, encouraging students to actively explore their own understanding and move beyond knowing-in-action; this can result in the contextual application of prior knowledge [11]. The tangible nature of the models fosters embodied cognition, where physical interaction supports learning and memory. Glenberg et al. emphasized that motor activity and embodiment can directly influence cognitive processing and memory retention [23]. Similarly, Kontra et al. demonstrated that active engagement with physical models enhances science learning outcomes compared to passive observation alone [24].

In addition, there was a substantial increase observed in participants’ scores for “interest in learning disaster medicine” and “willingness to participate in MCI management” following the exercise. Previous studies also revealed that participants found using miniatures in the simulation enjoyable and highly useful [6]. If students find exercises interesting, they are willing to exert the required effort, thereby facilitating the learning process [11].

These findings align with our participants’ feedback, which consistently emphasized the value of the 3D models in helping them "see the whole picture" and "feel the pressure" of real-time decision-making during mass casualty incidents. The models helped translate abstract principles of disaster medicine into concrete and relatable experiences. Importantly, the accessibility of 3D printing technology has improved significantly in recent years, making it feasible for training hospitals to develop customized, reusable models at a relatively low cost. It is also important to consider that the engaging nature of game-based learning itself may enhance motivation and interest, independent of the teaching content. Similar simulation-based training programs, such as the Terror and Disaster Surgical Care (TDSC®) course, have demonstrated that interactive formats can foster high learner engagement and preparedness in mass-casualty settings [25–27].

In summary, the integration of 3D tabletop exercises represents a promising educational strategy that leverages the strengths of spatial cognition, experiential learning, and modern fabrication technologies. Further research is warranted to examine long-term retention and the transferability of these skills into real clinical environments.

### Limitations

This study has several limitations. First, this study utilized a pre-post design, which is subject to inherent methodological biases. Among them, testing effects, recall bias, and social desirability bias were considered the most significant in our context. In the absence of a control group and objective assessments, it is difficult to determine whether the observed improvements were directly attributable to the intervention itself. However, it was not the aim of this study to demonstrate that the novel training tool is superior to existing educational methods. Our objective was to evaluate whether this tool could effectively enhance learners’ understanding of disaster medicine and key DMCCs. The findings of this study revealed a substantial improvement in post-training assessments, suggesting that the 3D-TTX is a feasible and effective alternative for disaster medicine education. This approach may serve as a valuable addition to the current educational toolkit, especially in settings where resources for large-scale drills or high-fidelity simulations are limited.

Second, we did not evaluate long-term retention of knowledge, interest, or willingness. All assessments were conducted immediately following the 3D-TTX. Although prior studies have reported high retention rates following TTX, the impact of longer follow-up intervals and repeated sessions remains to be explored.

Third, our assessment focused on only five DMCCs. Other relevant competencies were not evaluated, and it remains unclear whether the entire range of DMCCs can be effectively taught using this model. Future studies should investigate which competencies are best suited to this educational approach.

Fourth, all participants were recruited from a single medical school, which may limit the generalizability of the findings to other institutions or cultural contexts. Nevertheless, the training tool introduced in this study is cost-effective, reproducible, and potentially applicable in other educational settings, warranting further validation.

Finally, the game-based format itself may have contributed to the observed increases in student motivation and engagement. This inherent appeal could confound the effects of the instructional design, making it difficult to isolate the impact of the 3D-TTX structure from the novelty or attractiveness of the format. Even if the game-based format itself boosted interest and motivation, our results show that students still achieved comparable learning outcomes. This suggests game-based methods may be an effective and engaging alternative for disaster medicine education.

## Conclusions

An interactive TTX incorporating 3D miniature models, the 3D-TTX, appears to be a promising educational method for teaching core competencies in disaster medicine to senior medical students. This approach allows learners to engage with complex scenarios in a controlled, low-stress environment, facilitating active participation and decision-making.

Notably, the exercise was conducted without pre-reading or lectures, relying solely on students’ prior knowledge. While this design may have contributed to the perceived authenticity and learning gains, it may also have introduced uncertainty or frustration for some participants. Future implementations should explore whether minimal preparatory guidance or structured pre-briefing could enhance clarity and optimize the learning experience.

Despite these considerations, the exercise format demonstrated significant potential in increasing students’ interest in disaster medicine and their willingness to participate in future disaster response efforts. These findings support the educational value of experiential learning and suggest that 3D-TTXs can be feasibly integrated into undergraduate medical curricula and broader disaster preparedness training programs.

## Supplementary Information

Supplementary Material 1.

Supplementary Material 2.

## Data Availability

The datasets used and analyzed during the current study are available from the corresponding author on reasonable request.

## References

[1] Kasselmann N, Willy C, Domres BD, Wunderlich R, Back DA. Implementation of disaster medicine education in German medical schools – a nationwide survey. GMS J Med Educ. 2021;38(4):Doc79. 10.3205/zma001475 PMC8136347 34056068

[2] Wiesner L, Kappler S, Shuster A, DeLuca M, Ott J, Glasser E. Disaster training in 24 hours: evaluation of a novel medical student curriculum in disaster medicine. J Emerg Med. 2017;[Epub ahead of print]. 10.1016/j.jemermed.2017.12.008. 10.1016/j.jemermed.2017.12.008 29395693

[3] Winakor J, Janatpour ZC, West J. Medical student involvement in disasters: How can we effectively serve? Mil Med. 2021;usab181. 10.1093/milmed/usab181 10.1093/milmed/usab181 PMC8194527 33993269

[4] Datta R, Upadhyay K, Jaideep C. Simulation and its role in medical education. Med J Armed Forces India. 2012;68(2):167–72. 24623932 10.1016/S0377-1237(12)60040-9 PMC3862660

[5] Lennquist Montan K, Hreckovski B, Dobson B, Ortenwall P, Montan C, Khorram-Manesh A, et al. Development and evaluation of a new simulation model for interactive training of the medical response to major incidents and disasters. Eur J Trauma Emerg Surg. 2014;40(4):429–43. 26816238 10.1007/s00068-013-0350-y

[6] Idrose AM, Adnan WA, Villa GF, Abdullah AH. The use of classroom training and simulation in the training of medical responders for airport disaster. Emerg Med J. 2007;24(1):7–11. 17183034 10.1136/emj.2006.036202 PMC2658171

[7] Sarin RR, Cattamanchi S, Alqahtani A, Aljohani M, Keim M, Ciottone GR. Disaster Education: A Survey Study to Analyze Disaster Medicine Training in Emergency Medicine Residency Programs in the United States. Prehosp Disaster Med. 2017;32(4):368–73. 28318478 10.1017/S1049023X17000267

[8] Sena A, Forde F, Yu C, Sule H, Masters MM. Disaster Preparedness Training for Emergency Medicine Residents Using a Tabletop Exercise. MedEdPORTAL. 2021;17:11119. 33768151 10.15766/mep_2374-8265.11119 PMC7970644

[9] Castro Delgado R, Fernandez Garcia L, Cernuda Martinez JA, Cuartas Alvarez T, Arcos GP. Training of Medical Students for Mass Casualty Incidents Using Table-Top Gamification. Disaster Med Public Health Prep. 2022;17:e255. 36128647 10.1017/dmp.2022.206

[10] Sarpy SA, Warren CR, Kaplan S, Bradley J, Howe R. Simulating public health response to a severe acute respiratory syndrome (SARS) event: a comprehensive and systematic approach to designing, implementing, and evaluating a tabletop exercise. J Public Health Manag Pract. 2005;11(6):S75–82. 10.1097/00124784-200511001-00013 16205548

[11] Angafor GN, Yevseyeva I, He Y. Game‐based learning: A review of tabletop exercises for cybersecurity incident response training. Security and Privacy. 2020;3(6):e126.

[12] Beigzadeh A, Rahimi M. The use of games in medical education. Res Dev Med Educ. 2015;4(1):1–2.

[13] Chi CH, Chao WH, Chuang CC, Tsai MC, Tsai LM. Emergency medical technicians’ disaster training by tabletop exercise. Am J Emerg Med. 2001;19(5):433–6. 11555806 10.1053/ajem.2001.24467

[14] Savoia E, Biddinger PD, Fox P, Levin DE, Stone L, Stoto MA. Impact of tabletop exercises on participants’ knowledge of and confidence in legal authorities for infectious disease emergencies. Disaster Med Public Health Prep. 2009;3(2):104–10. 19491605 10.1097/DMP.0b013e3181a539bc

[15] BBC News Chinese. (2016, July 7). Taiwan Formosa Fun Coast explosion: 499 injured, 202 in serious condition. BBC News. https://www.bbc.com/zhongwen/trad/china/2016/07/160707_taiwan_blast

[16] Subbarao I, Lyznicki JM, Hsu EB, Gebbie KM, Markenson D, Barzansky B, et al. A consensus-based educational framework and competency set for the discipline of disaster medicine and public health preparedness. Disaster Med Public Health Prep. 2008;2(1):57–68. 18388659 10.1097/DMP.0b013e31816564af

[17] Walsh L, Subbarao I, Gebbie K, Schor KW, Lyznicki J, Strauss-Riggs K, et al. Core competencies for disaster medicine and public health. Disaster Med Public Health Prep. 2012;6(1):44–52. 22490936 10.1001/dmp.2012.4

[18] Schultz CH, Koenig KL, Whiteside M, Murray R, Force NSA-HDCCT. Development of national standardized all-hazard disaster core competencies for acute care physicians, nurses, and EMS professionals. Annals of emergency medicine. 2012;59(3):196–208. e1. 10.1016/j.annemergmed.2011.09.003 21982151

[19] Federal Emergency Management Agency. (n.d.). Homeland Security Exercise and Evaluation Program (HSEEP). U.S. Department of Homeland Security. https://www.fema.gov/emergency-managers/national-preparedness/exercises/hseep

[20] Scott LA, Maddux PT, Schnellmann J, Hayes L, Tolley J, Wahlquist A. High fidelity multi-actor emergency preparedness training for patient care providers. Am J Disaster Med. 2012;7(3):175. 23140061 10.5055/ajdm.2012.0093 PMC3678946

[21] Ma D, Shi Y, Zhang G, Zhang J. Does theme game-based teaching promote better learning about disaster nursing than scenario simulation: A randomized controlled trial. Nurse Educ Today. 2021;103:104923. 33962185 10.1016/j.nedt.2021.104923

[22] Whitney RE, Burke RV, Lehman-Huskamp K, Arora G, Park DB, Cicero MX. On shaky ground: learner response and confidence after tabletop earthquake simulation. Pediatr Emerg Care. 2016;32(8):520–4. 26999584 10.1097/PEC.0000000000000681

[23] Glenberg AM, Gutierrez T, Levin JR, Japuntich S, Kaschak MP. Activity and imagined activity can enhance young children’s reading comprehension. J Educ Psychol. 2004;96(3):424–36. 10.1037/0022-0663.96.3.424.

[24] Kontra C, Lyons DJ, Fischer SM, Beilock SL. Physical experience enhances science learning. Psychol Sci. 2015;26(6):737–49. 10.1177/0956797615569355. 25911125 10.1177/0956797615569355

[25] Hoth P, Roth J, Bieler D, Friemert B, Franke A, Paffrath T, et al. Education and training as a key enabler of successful patient care in mass-casualty terrorist incidents. Eur J Trauma Emerg Surg. 2023;49(2):595–605. 10.1007/s00068-023-02232-w. 36810695 10.1007/s00068-023-02232-w PMC10175327

[26] Bieler D, Franke A, Blätzinger M, Hofmann M, Sturm J, Friemert B, et al. Evaluation of the Terror and Disaster Surgical Care course. Eur J Trauma Emerg Surg. 2020;46(4):709–16. 10.1007/s00068-020-01418-w. 32749506 10.1007/s00068-020-01418-w

[27] Achatz G, Bieler D, Franke A, Friemert B. Terror preparedness as a service of general interest: the Terror and Disaster Surgical Care (TDSC®)-course. Eur J Trauma Emerg Surg. 2020;46(4):671–2. 10.1007/s00068-020-01454-6. 32803381 10.1007/s00068-020-01454-6

