# Supplementary material for: Teaching mass casualty incident management to senior medical students by three-dimensional tabletop exercise without lecture
Source: BMC Med Educ. 2025 Jun 5;25:846. doi: 10.1186/s12909-025-07434-x (PMC12142820; doi:10.1186/s12909-025-07434-x)
Supplement: Supplementary file 1 — Supplementary Material 1. [file 12909_2025_7434_MOESM1_ESM.docx]

**Master Scenario Events List (MSEL)**

| Event | Date | Time | Category | Inject Mode | Synopsis | From | To | Message | Expected Action | Responsible Organization | Comment |
| --- | --- | --- | --- | --- | --- | --- | --- | --- | --- | --- | --- |
| Inject 1 | 04/20 | 17:10 | Inject | Verbal / Slide | Fire Department informs the ED of a bombing... | City Fire Department | Emergency Department | Fire Department informs the ED of a bombing at Taipei Main Station. Possible mass casualties expected. | Recognize incident, begin internal notification | NTUH Emergency Department | Recognition, Notification, and Initiation |
| Inject 2 | 04/20 | 17:12 | Inject | Verbal / Slide / miniatures | Hospital personnel receive alert and begin to gath... | ED Director | ED Staff | Hospital personnel receive alert and begin to gather; roles need to be assigned. | Initiate command structure and assign roles | NTUH Emergency Department | Incident Management System |
| Inject 3 | 04/20 | 17:20 | Inject | Verbal / Slide / miniatures | Treatment zones planned and designated based on ex... | ED Director | ED Staff | Treatment zones planned and designated based on expected surge. | Designate triage, treatment, and waiting areas | NTUH Emergency Department | Incident Management System |
| Inject 4 | 04/20 | 17:30 | Inject | Verbal / Slide / miniatures | Taxi arrives with 3 walking wounded who self-prese... | Civilians | Triage Nurse | Taxi arrives with 3 walking wounded who self-present. | Triage and register walk-in patients | NTUH Emergency Department | Patient Triage |
| Inject 5 | 04/20 | 17:51 | Inject | Verbal / Slide / miniatures | Ambulance brings 15 casualties including severe bu... | EMS | ED | Ambulance brings 15 casualties including severe burns and trauma. | Activate surge capacity and initiate triage | NTUH Emergency Department | Surge Capacity and Capability |
| Inject 6 | 04/20 | 18:00 | Inject | Verbal / Slide | Hospital leadership inquires about activating addi... | Hospital Director | ED Commander | Hospital leadership inquires about activating additional emergency measures. | Consider hospital-wide alert or activation of emergency ops | NTUH Emergency Department | Surge Capacity and Capability |
| Inject 7 | 04/20 | 18:05 | Inject | Verbal / Slide / miniatures | Additional staff arrive for support roles. | HR/Support | ED | Additional staff arrive for support roles. | Assign newly arrived staff to roles | NTUH Emergency Department | Surge Capacity and Capability |
| Inject 8 | 04/20 | 18:10 | Inject | Verbal / Slide / miniatures | Family brings in deceased victim and demands resus... | Family | ED Staff | Family brings in deceased victim and demands resuscitation. | Assess and explain death protocols | NTUH Emergency Department | Patient Triage |
| Inject 9 | 04/20 | 18:30 | Inject | Verbal / Slide / miniatures | Initially walking patient collapses unexpectedly. | Patient | ED Staff | Initially walking patient collapses unexpectedly. | Initiate emergency reassessment and stabilize | NTUH Emergency Department | Patient Triage |
| Inject 10 | 04/20 | 18:40 | Inject | Verbal / Slide / miniatures | Multiple family members enter ED looking for loved... | Family | ED Info Desk | Multiple family members enter ED looking for loved ones. | Manage visitor flow and provide information | NTUH Emergency Department | Incident Management System |
| Inject 11 | 04/20 | 18:45 | Inject | Verbal / Slide / miniatures | Ambulance arrives with pulseless patient, no visib... | EMS | ED Resuscitation Team | Ambulance arrives with pulseless patient, no visible trauma. | Attempt resuscitation, log arrival | NTUH Emergency Department | Triage |
| Inject 12 | 04/20 | 18:50 | Inject | Verbal / Slide | Phone inquiries from family members about patient ... | Family | ED Clerk | Phone inquiries from family members about patient status. | Provide limited, verified updates | NTUH Emergency Department | Incident Management System |
| Inject 13 | 04/20 | 18:55 | Inject | Verbal / Slide / miniatures | Media personnel enter ED attempting interviews. | Journalists | ED Security | Media personnel enter ED attempting interviews. | Enforce access control and notify administration | NTUH Emergency Department | Incident Management System |
| Inject 14 | 04/20 | 19:40 | Inject | Verbal / Slide | Public health authority requests status update fro... | DOH | ED | Public health authority requests status update from ED. | Provide accurate situation report | NTUH Emergency Department | Notification |
| Inject 15 | 04/20 | 20:20 | Inject | Verbal / Slide / miniatures | Media demand press conference outside hospital. | Press | Hospital Spokesperson | Media demand press conference outside hospital. | Coordinate with admin and PR office | NTUH Emergency Department | Incident Management System |
| Inject 16 | 04/20 | 21:30 | Inject | Verbal / Slide | Fire Department informs that all victims are trans... | City Fire Department | ED | Fire Department informs that all victims are transported. | Confirm patient intake is complete | NTUH Emergency Department | Notification |
| Inject 17 | 04/20 | 23:30 | Inject | Verbal / Slide / miniatures | ED now only has 5 mild observation cases remaining... | ED Report | ED Command | ED now only has 5 mild observation cases remaining. | Begin demobilization and documentation | NTUH Emergency Department | Recovery and Demobilization |
| Inject 18 | 04/20 | 23:45 | Inject | Verbal / Slide | Hospital administration asks about after-action pr... | Admin | ED Leadership | Hospital administration asks about after-action process. | Plan debrief and begin after-action review | NTUH Emergency Department | Recovery and Demobilization |
