# Supplementary material for: Teaching mass casualty incident management to senior medical students by three-dimensional tabletop exercise without lecture
Source: BMC Med Educ. 2025 Jun 5;25:846. doi: 10.1186/s12909-025-07434-x (PMC12142820; doi:10.1186/s12909-025-07434-x)
Supplement: Supplementary file 2 — Supplementary Material 2. [file 12909_2025_7434_MOESM2_ESM.docx]

Pre- and Post- exercise survey

**Age**: ______

**Gender:**

- Male
- Female

**Previous disaster training experience**

- None
- Once
- Twice
- >3 times

**How interested are you in learning disaster medicine?**

- 1 = very uninterested
- 2
- 3
- 4
- 5 = very interested

**How willing are you to participate in managing mass casualty incidents?**

- 1 = very unwilling
- 2
- 3
- 4
- 5 = very willing

**Knowledge assessment:**

Part A.: Incident management system

**A-1 In the Incident Command System (ICS), which of the following job assignments is the most 'inappropriate'?**

(A.) Triage of patients: Operations Section

(B.) Establishment of the command center communication system: Planning Section

(C.) Providing meals for emergency personnel: Logistics Section

(D.) Registering attendance of emergency personnel: Finance/Administration Section

**A-2 In the command staff of the ICS, which of the following position and job pairings is 'incorrect'?**

(A.) Incident Commander: understanding the current situation of the incident

(B.) Public Information Officer: releasing the current response status of the hospital to the media

(C.) Liaison Officer: reporting the hospital's current response status to the Health Bureau

(D.) Safety Officer: setting up barriers and controlling the media from entering the emergency room

**A-3 Regarding the coordination and assignment of tasks in the ICS, which of the following is correct?**

(A.) Information needed by the Public Information Officer is provided by the Operations Section.

(B.) If a member of the Operations Section is injured, they are treated by the Logistics Section.

(C.) If a member of the Planning Section is hungry, meals are provided by the Finance/Administration Section.

(D.) The response objectives set by the Incident Commander are verified for safety by the Liaison Officer.

**A-4 Which of the following descriptions about ICS regulations is the most 'incorrect'?**

(A.) The number of people in a group is usually controlled within 3 to 7 people.

(B.) Commands in the ICS are transmitted top-down only, with no horizontal transmission.

(C.) If a new task within a group is not assigned, it is managed by the Incident Commander.

(D.) Units under each section can be activated in stages as needed.

Part B: Recognition, notification, and initiation

**B-1 While working in the emergency department of this hospital, which of the following is the least likely to be an indication of a mass casualty incident?**

(A.) Patients continuously coming to register

(B.) Notification from the Fire Department Emergency and Rescue Command Center of a mass casualty incident

(C.) Many patients indicating they come from the same accident scene

(D.) Seeing news reports of a major accident near the hospital

**B-2 While working in the emergency department of this hospital, upon being informed of a mass casualty incident, which of the following response actions is the most inappropriate?**

(A.) Trying to understand the incident situation through various means

(B.) Informing the attending physician on duty

(C.) Notifying the Department of Emergency Medicine director

(D.) Informing the switchboard to broadcast a "Code Brown 1"

**B-3 When you receive a notification from the Fire Department Emergency and Rescue Command Center that the hospital is facing a mass casualty incident, which of the following information is the least necessary to inquire about?**

(A.) The ratio of minor to severe injuries at the scene

(B.) The emergency response personnel mobilized at the scene

(C.) The estimated time of arrival at the hospital

(D.) The details of the event causing the mass casualties

**B-4 Emergency management is divided into preparation, mitigation, response, and recovery phases. Which of the following tasks is not part of the response phase for a mass casualty incident in the Department of Emergency Medicine?**

(A.) Planning the response organization structure

(B.) Broadcasting the incident code via the switchboard

(C.) Assembling the hospital's emergency response personnel

(D.) Briefing the emergency response personnel

Part C: Patient triage

**C-1 Which of the following correctly describes the difference between mass casualty triage and general emergency room (ER) triage?**

(A.) Mass casualty triage is faster.

(B.) The five-level triage system used in the ER has been proven to be more accurate for prognosis assessment.

(C.) Mass casualty triage requires the use of more tools (e.g., blood pressure monitors).

(D.) General ER triage uses fewer parameters (e.g., blood pressure, heart rate) for classification.

**C-2 According to the START (simple triage and rapid treatment) principles, which of the following patient and triage category pairings is incorrect?**

(A.) Head trauma, unconscious, shallow and rapid breathing, palpable radial pulse – Black

(B.) Swollen and deformed left forearm, normal breathing, palpable radial pulse, and able to walk – Green

(C.) Abdominal pain, non-palpable radial pulse, normal breathing and consciousness – Red

(D.) Swollen and deformed thigh, palpable radial pulse, normal breathing and consciousness – Yellow

**C-3 Which of the following is the least accurate reason for the need for triage in a mass casualty incident?**

(A.) Providing limited medical resources to the greatest number of patients

(B.) Assessing the severity of injuries

(C.) Identifying critical patients

(D.) Assigning patients to appropriate treatment areas

**C-4 When a patient's condition seems to deteriorate after triage but before being treated, which of the following actions is the most appropriate?**

(A.) Immediately report to the Incident Commander

(B.) Re-triage the patient

(C.) Ask the area supervisor to handle it as soon as possible

(D.) Administer oxygen and saline to the patient

Part D: Surge capacity / capability

**D-1 In a mass casualty incident, which of the following is incorrect regarding the differences in patient management compared to general ER situations?**

(A.) Whether to resuscitate a patient with pre-hospital cardiac arrest is unrelated to the availability of clinical resources.

(B.) Reducing some treatments for less severe patients.

(C.) Increasing the extent of self-care by patients.

(D.) Shortening some administrative procedures to speed up patient management.

**D-2 Which of the following is incorrect regarding space planning when managing a mass casualty incident?**

(A.) When the number of casualties is still uncertain, consider flexible use of space and possibly expanding the area.

(B.) Utilizing areas not usually designated for patients, such as the hospital lobby.

(C.) Since it affects the rights of existing patients, original ER patient beds should not be moved.

(D.) When choosing spaces, pay attention to the availability of power and oxygen.

**D-3 Which of the following is not a task during a mass casualty incident response?**

(A.) Calming families and assisting with inquiries about casualties

(B.) Receiving media and releasing information to the public

(C.) Reporting the incident situation to local health department

(D.) Employee education and training exercises

**D-4 To increase the hospital's capacity to manage a mass casualty incident, which of the following is not an appropriate response measure?**

(A.) Postponing originally scheduled but non-urgent surgeries

(B.) Notifying the fire department of full capacity to prevent more patients from being sent to the hospital

(C.) Accelerating patient admission procedures to prevent overcrowded

(D.) Calling back off-duty staff to increase emergency response manpower

Part E: Recovery and demobilization

**E-1 Which of the following is the most important consideration for initiating the demobilization procedure?**

(A.) Emergency responders at the incident scene notify that there are no more casualties.

(B.) The Fire Department Emergency and Rescue Command Center notifies that there are no more casualties at the scene.

(C.) No casualties have been treated in the past hour.

(D.) The number and severity of casualties in the emergency department can be managed using standard ER protocol.

**E-2 When initiating the demobilization procedure, which of the following is the least prioritized for demobilization?**

(A.) Administrative staff

(B.) Incident Commander

(C.) Ward nurses

(D.) Safety Officer

**E-3 Which of the following is not a task during the recovery phase of a mass casualty incident in the Department of Emergency Medicine?**

(A.) Rescheduling surgeries that were previously canceled or postponed

(B.) Collecting and organizing documents and data used during the response

(C.) Restocking resources used during the response and returning equipment to its place

(D.) Convening the crisis management committee to draft a response plan

**E-4 An important task during the recovery phase is compiling the response process into a report as a reference for future incident management. Which of the following is the least appropriate to include in the report?**

(A.) Personnel to be reprimanded

(B.) Summary of the incident from beginning to end

(C.) Summary of the response actions

(D.) Actions that were handled appropriately
